# Mutational analysis of *BRCA1* and *BRCA2* in hereditary breast and ovarian cancer families from Asturias (Northern Spain)

**DOI:** 10.1186/1471-2407-13-243

**Published:** 2013-05-17

**Authors:** Pilar Blay, Iñigo Santamaría, Ana S Pitiot, María Luque, Marta G Alvarado, Ana Lastra, Yolanda Fernández, Ángeles Paredes, José MP Freije, Milagros Balbín

**Affiliations:** 1 Unidad de Cáncer Familiar, Servicio de Oncología Médica, Instituto Universitario de Oncología del Principado de Asturias (IUOPA), Hospital Universitario Central de Asturias (HUCA), Calle de Celestino Villamil, Oviedo 33006, Spain; 2 Laboratorio de Oncología Molecular, IUOPA, Laboratorio de Medicina, HUCA, Calle de Celestino Villamil, Oviedo 33006, Spain; 3 Servicio de Oncología Médica, IUOPA, HUCA, Calle de Celestino Villamil, Oviedo 33006, Spain; 4 Dpto. de Bioquímica y Biología Molecular, IUOPA, Universidad de Oviedo, Calle de Fernando Bongera, Oviedo 33006, Spain

**Keywords:** Hereditary breast and ovarian cancer, *BRCA1*, *BRCA2*, Recurrent mutations, Asturian population

## Abstract

**Background:**

The prevalence of *BRCA1* and *BRCA2* mutations in Spain is heterogeneous and varies according to geographical origin of studied families. The contribution of these mutations to hereditary breast and ovarian cancer has not been previously investigated in Asturian populations (Northern Spain).

**Methods:**

In the present work, 256 unrelated high-risk probands with breast and/or ovarian cancer from families living in Asturias were analyzed for the presence of a *BRCA1* or *BRCA2* gene mutation from October 2007 to May 2012. The entire coding sequences and each intron/exon boundaries of BRCA1/2 genes were screened both by direct sequencing and Multiplex Ligation-dependent Probe Amplification (MLPA).

**Results:**

A total of 59 families (23%) were found to carry a pathogenic germ line mutation, 39 in *BRCA1* and 20 in *BRCA2*. Twenty nine additional families (12%) carried an unknown significance variant. We detected 28 distinct pathogenic mutations (16 in *BRCA1* and 12 in *BRCA2*), of which 3 mutations in *BRCA1* (c.1674delA, c.1965C>A and c.2900_2901dupCT) and 5 in *BRCA2* (c.262_263delCT, c.2095C>T, c.3263dupC, c.4030_4035delinsC, c.8042_8043delCA) had not been previously described.

The novel mutations c.2900_2901dupCT in *BRCA1* and c.4030_4035delinsC in *BRCA2* occurred in 8 and 6 families respectively and clustered in two separated small geographically isolated areas suggesting a founder effect. These 2 mutations, together with the Galician *BRCA1* mutation c.211A>G (9 families), and the common *BRCA1* mutation c.3331_3334delCAAG (6 families), account for approximately 50% of all affected families. By contrast, very frequent mutations in other Spanish series such as the *BRCA1* Ashkenazi founder mutation c.68_69delAG, was found in only one family.

**Conclusions:**

In this study we report the *BRCA1* and *BRCA2* spectrum of mutations and their geographical distribution in Asturias, which largely differ from other areas of Spain. Our findings may help design a first step recurrent mutation panel for screening high-risk breast and/or ovarian cancer families from this specific area.

## Background

Breast cancer is the most frequently diagnosed and the leading cause of cancer death among females, accounting for 23% of the total cases and 14% of the cancer deaths [1]. While most tumors are sporadic, about 5 to 10% are caused by germ line mutations in certain genes [2]. *BRCA1* (MIM 113705) [3] and *BRCA2* (MIM 600185) [4] genes are responsible for approximately 20-40% of inherited breast cancer [5,6]. Women carrying germ line mutations in these genes have a high lifetime risk of developing both breast and ovarian cancer [7]. Testing these high-penetrant genes can make it feasible to identify individuals at risk as candidates for surveillance programs [8].

Prevalence of *BRCA1* or *BRCA2* germ line mutations varies considerably among ethnic groups, and in some countries, founder mutations are responsible for a significant proportion of breast cancer cases. Specific mutations have been described, for example, among Ashkenazi Jews, in Iceland, and in several other countries where isolated populations exists [9]. Mutational analysis of *BRCA1* and *BRCA2* in hereditary breast and ovarian cancer families have also been reported in several Spanish regions, showing a heterogeneous prevalence of recurrent mutations according to the geographical area [10]. However there are to date no comprehensive studies in the Asturian population, a geographically isolated area in the North of Spain.

In this article, we present mutation detection data corresponding to a set of 256 high-risk families living in Asturias, analyzed by direct sequencing and MLPA. Thirty-nine families were found to have a mutation in *BRCA1* gene and 20 in *BRCA2*. We report the finding of 8 novel mutations and the clustered geographical distribution of two of them which may be founder mutations.

## Methods

### Study population

Patients were referred to the Familial Cancer Unit at Hospital Universitario Central de Asturias (HUCA) for genetic counseling by physicians between October 2007 and May 2012. Information about the number and type of cancers, age of diagnosis, age of death or current age and geographical origin were collected from each family. Genetic testing was offered to affected individuals from high-risk families meeting any of the following criteria:

A. Three or more family members with breast and/or ovarian cancer.

-- A. Br: only breast cancer.

-- A. Ov: at least one ovarian cancer.

B. Two family members with ovarian cancer.

C. One family member with ovarian cancer and one with breast cancer.

D. One family member with a male breast cancer and one or more with breast and/or ovarian cancer.

E. Two family members with breast cancer, before the age of 50.

F. One family member with bilateral breast cancer and one with breast cancer, at least one before the age of 50.

G. Single affected individual with either:

-- Ga: bilateral breast cancer, first diagnosed before the age of 40, or

-- Gb: breast or ovarian cancer diagnosed before the age of 30, or

-- Gc: breast and ovarian cancer

H. Affected probands from families close to fulfill any of the above criteria. We have included in this group affected probands with a history suggestive of hereditary breast and ovary cancer who do not fulfill completely any of the above inclusion criteria. This included, for instance, probands from small families with two affected relatives older than 50 years of age with triple negative breast cancer.

A total of 256 families fulfilled any of the selection criteria. The study was approved by the hospital ethical committee. All tested individuals provided a signed informed consent following appropriate genetic counseling. Blood samples were obtained from at least one affected member. DNA and RNA were obtained from peripheral blood from all studied members of the family. Index case was first analyzed for large genomic rearrangements and then for sequence mutations. Genomic DNA was obtained after red blood cell lyses using DNAzol method (MRC, USA). RNA was purified from leucocytes by using TriReagent solution (Ambion, Life Technologies).

### Large genomic rearrangements of *BRCA1* and *BRCA2*

*BRCA1* and *BRCA2* large genomic rearrangements were assayed by MLPA (multiplex ligation-dependent probe amplification) by using P002 and P087 kits for *BRCA1* gene and P045 kit for *BRCA2* gene (MRC-Holland), following essentially the instructions of the manufacturer. Amplified products were electrophoretically separated in an ABIPrism310 genetic analyzer and interpreted using the *GeneMapper* 4.0 software (Applied Biosystems). For normalizing the data, at least two genomic DNA samples obtained from peripheral blood cells of healthy donors were always run as controls in each analysis. Quantification of the results, measuring peak areas, was performed by using an Excel calculation sheet. The final allele dosage for each allele was obtained from the following formula:

(“n” peak area from sample / “n” peak area from control) divided by (∑ reference peak areas from sample / ∑ reference peak areas from control).

Normal values were considered when this ratio was between 0.8 and 1.2.

### Mutation analysis of *BRCA1* and *BRCA2*

Mutational screening of *BRCA1* and *BRCA2* genes was performed by direct sequencing of each exon and 20 bp exon-intron boundaries, by standard Sanger sequencing using BigDye™ terminator sequencing kits (Applied BioSystems). Sequence analyses for some index cases were performed at Sistemas Genómicos SL (Valencia, Spain) or at Imegen (Parque Científico, Universidad de Valencia, Spain).

Reference sequences used for *BRCA1* and *BRCA2* analyses were GenBank NM_007294.2 (*BRCA1*) and NM_000059.3 (*BRCA2*). Mutation nomenclature is described according to Human Genome Variation Society (v2.0) (http://www.hgvs.org) [11].

## Results and discussion

### Frequency of families with *BRCA1* and *BRCA2* pathogenic mutations according to the inclusion criteria

We identified 59 families carrying a pathogenic mutation, 39 in *BRCA1* and 20 in *BRCA2,* which represent 23% of all screened families (Table 1). The highest mutation detection rate in *BRCA1* was observed in families with at least one ovarian cancer (groups A.Ov., B, C, and Gc.) and in families with a single case of bilateral breast cancer diagnosed before the age of 40 (group Ga). Regarding *BRCA2*, the highest detection rate was observed in families with one breast cancer and one ovarian cancer and in families with a male breast cancer (groups C and D), while mutations were not found in any of the groups with single cases (group G) or two ovarian cancers (group B). Three out of 11 families with a male breast cancer (group D) carried a pathogenic mutation, 1 in *BRCA1* and 2 in *BRCA2*. These mutation detection rates are comparable to previously reported populations. No pathogenic mutations were found in 9 women with early onset breast cancer without family history (group Gb). Although this is an inclusion criterion in all clinical guides, other authors have also found very low mutation rate in this group [12,13]. On the other hand, we identified a deleterious mutation in 2 out of 19 affected probands (10.5%) who did not fully meet our present inclusion criteria for testing. Within this group (H), a *BRCA1* deleterious mutation was found in a family with two affected first degree relatives diagnosed with breast cancer over the age of 50, having one of them a “triple negative” phenotype in the tumor. The other proband was a 47-year-old woman, diagnosed with ovarian cancer and with no family history of cancer, in whom a *BRCA2* pathogenic mutation was identified.

**Table 1:** **Frequency of families with *BRCA1/2 *pathogenic mutations according to the inclusion criteria**

**Family type**	**Tested families**	**BRCA1**	**(%)**	**BRCA2**	**(%)**	**BRCA1/2**	**(%)**
A. Br. (3 or >, only Br)	80	5	(6.3)	6	(7.5)	11	(13.8)
A. Ov. (3 or >, at least 1 Ov)	48	16	(33.3)	4	(8.3)	20	(41.7)
B. 2 Ov	2	1	(50.0)	0	(0.0)	1	(50.0)
C. 1 Br + 1 Ov	33	9	(27.3)	4	(12.1)	13	(39.4)
D. 1 mBr and Br/Ov	11	1	(9.1)	2	(18.2)	3	(27.3)
E. 2 Br<50	35	3	(8.6)	2	(5.7)	5	(14.3)
F. bBr and other <50	13	1	(7.7)	1	(7.7)	2	(15.4)
G. a. 1 bBr<40	2	1	(50.0)	0	(0.0)	1	(50.0)
G. b. 1 Br<30	9	0	(0.0)	0	(0.0)	0	(0.0)
G. c. 1 BrOv	4	1	(25.0)	0	(0.0)	1	(25.0)
H. borderline	19	1	(5.3)	1	(5.3)	2	(10.5)
Total	256	39	(15.2)	20	(7.8)	59	(23.0)

Br, breast cancer; Ov, ovarian cancer; mBr, male breast cancer; bBr, bilateral breast cancer.

### *BRCA1* deleterious mutations

Analysis of the *BRCA1* gene revealed 16 distinct germ line mutations with predicted deleterious effects on the BRCA1 protein in 39 families (Table 2). Among these pathogenic mutations, 3 are novel and have not yet been reported in the Breast Cancer Information Core, BIC (http://research.nhgri.nih.gov/bic/). One of these novel *BRCA1* mutations, c.2900_2901dupCT (p.Pro968Leufs), was shared by 8 apparently unrelated families and it accounted for 20% of *BRCA1* mutated families. This mutation is responsible for 7 breast and 7 ovarian cancer cases. Mean age of diagnosis was 49 years (range 33–78) for breast and 53 years (range 41–82) for ovarian cancer. Clinical and pathological characteristics of these families are shown in Table 3. The two remaining *BRCA1* novel mutations c.1674delA (p.Gly559Valfs) and c.1965C>A (p.Tyr655*) were found only in one family each.

**Table 2:** **Germ line *BRCA1 *pathogenic mutations in breast and ovarian cancer families from Asturias**

**Exon**	**BIC nomenclature**	**HGVS nomenclature**	**Predicted effect**	**N families**	**Families origen**	**Comments**
2	185delAG	c.68_69delAG	p.Glu23Valfs	1	Asturias	Ashkenazi Founder
5	330A>G	c.211A>G	p.Arg71Gly	9	Galicia	Galician Founder
8	589delCT	c.470_471delCT	p.Ser157*	3	Asturias	Spain/Worldwide
11	910del4	c.791_794delGTTC	p.Ser264Metfs	1	Castilla/León	Spain
11		c.1674delA	p.Gly559Valfs	1	Asturias	Novel
11	1806C>T	c.1687C>T	p.Gln563*	1	Castilla/León	Spain/Sweden Founder
11		c.1965C>A	p.Tyr655*	1	Asturias	Novel
11		c.2900_2901dupCT	p.Pro968Leufs	8	Asturias	Novel
11	3450del4	c.3331_3334delCAAG	p.Gln1111Asnfs	6	Several	Spain/Worldwide
11	3808T>G	c.3689T>G	p.Leu1230*	1	Castilla/León	Spain/Portugal
11	3889delAG	c.3770_3771delAG	p.Glu1257Glyfs	2	Several	Spain/Worldwide
11	4184del4	c.4065_4068delTCAA	p.Asn1355Lysfs	1	Asturias	Worldwide
18	5236G>C	c.5117G>C	p.Gly1706Ala	1	Asturias	Spain/Europe
	**LARGE GENOMIC REARRANGEMENTS**					
1 -24		BRCA1 del Ex 1-24		1	Asturias	Spain/Europe
1-13		BRCA1 del Ex 1-13		1	Asturias	Spain/Europe
20		BRCA1 del Ex 20		1	Asturias	Europe

**Table 3:** **Clinic pathological characteristics of tumors from individuals carrying the *BRCA1 *mutation c.2900_2901 dupCT (p.Pro968Leufs)**

**Family.individual**	**Sex**	**Age**	**Tumor**	**Histopathology**	**Stage**	**Grade**	**ER**	**PgR**	**HER2**
42.1	F	52	Ov	Endometrioid	IIIC	-	-	-	-
42.5	F	82	Ov	Serous	IIIC	-	-	-	-
72.1	F	54	Ov	Undifferentiated/Mucinous	IC	3	-	-	-
72.2	F	33	Br	Undifferentiated	IIIA	3	Neg	Neg	Neg
215.1	F	41	Ov	Serous	IIIC	3	-	-	-
263.1	F	56	Ov	Serous	IIIB	2	-	-	-
458.1	F	43	Br	IDC	II	3	Neg	Neg	Neg
458.2	F	51	Br	IDC	III	3	Neg	Neg	Neg
572.1	F	41	Ov	Serous	IIIC	-	-	-	-
704.1	F	49	Br	IDC	IIIA	-	Neg	Neg	-
828.1	F	48	Br	IDC	I	-	Pos	Pos	-
		78	Br	IDC	IA	3	Pos	Pos	Neg
828.2	F	42	Br	IDC	IIA	3	Pos	Pos	-
828.3	F	45	Ov	-	-	-	-	-	-

Age age of cancer diagnosis, F female, Br breast cancer, Ov ovarian cancer, IDC invasive ductal carcinoma, ER estrogen receptor status, PR progesterone receptor status, Her2 Her2Neu status, - not applicable or no data, Neg negative, Pos positive.

Among previously described mutations in *BRCA1*, the most common was the mutation c.211A>G (p.Arg71Gly), which was present in 9 families in our series (23%). This mutation is a founder mutation in Galicia, a Spanish region located just alongside western Asturias, where it represents 50% of all mutations [14,15], but it is almost absent in other Spanish populations [10]. All families found in our study to carry this mutation were either from Galicia or had Galician ancestors. Another common mutation, the c.3331_3334delCAAG (p.Gln1111Asnfs), was found in 6 families, thus representing 15% of *BRCA1* mutated families. It was first described in 1996 in a Canadian family [16] and thereafter in populations from all over the word. More recently an haplotype analysis performed in Hispanic populations living in Colombia suggested that this mutation has arisen from a common ancestor and that could represent a founder effect of Spanish origin [17]. This common mutation has 40 records in the BIC database, and has been reported in other regions of Spain [18,19]. The c.470_471delCT (p.Ser157*) mutation in exon 8 was detected in 3 families, being also reported in other Spanish [20,21], Chinese [22] and in Portuguese populations [23].

Other previously reported mutations were less frequent in our study. Thus, the nonsense mutation 1687C>T (p.Gln563*) was found in one family originally from León, Spain. Although this mutation was first described in 1996 in a Swedish population where it was considered to have a founder effect [24], it has also been reported in the Spanish area where this family came from (Castilla-León) [21]. From the same region were also two families carrying the mutations c.791_794delGTTC (p.Ser264Metfs) and c.3689T>G (p.Leu1230*). Both mutations have been previously reported in Spanish populations [15,20], as well as the mutation c.3770-3771delAG (p.Glu1257Glyfs), found in two families from our study.

Notably, the frame shift mutation c.4065_4068delTCAA (p.Asn1355Lysfs), which is one of the most frequently reported (133 entries in BIC) [25,26], was only found in one of our families and has not been previously reported in Spain. The Ashkenazi founder mutations c.68_69delAG (p.Glu23Valfs) was found in only one family, while it is the most frequent *BRCA1* alteration found in other Spanish series [20]. Finally, we found the missense mutation c.5117G>C (p.Gly1706Ala) in one family. Although this mutation is still annotated as one with unknown clinical importance in the BIC database, some studies support that it could be pathogenic [20,27].

### *BRCA1* large genomic deletion

An MLPA-based search for large genomic rearrangements was performed in all index cases. This approach revealed that three of them were positive for the presence of a large deletion in the *BRCA1* gene. One family presented heterozygous deletions for all the probes specific for *BRCA1*, from exon 1 to exon 24, revealed with two different MLPA kits (P002B and p084). The index case was a 56 years old woman with ovarian cancer. Her mother had also ovarian cancer and a sister was affected of breast cancer. After a complete study of all available members of the family, we could confirm that the deletion segregated with the disease. *BRCA1* complete deletions have only been previously reported in 3 families, being a *de novo* mutation in one of them [28-30].

A second case showed an MLPA profile suggesting heterozygous deletions in probes specific for exons 1 to 13. The pattern was displayed with probes from P002B kit and confirmed with P082 MLPA kit exons 1–13. The deletion was identified in a woman with ovarian cancer at the age of 64 who had had a bilateral breast cancer at ages 42 and 61 respectively. Her sister had also had an ovarian cancer at the age of 33. This mutation has also been described in another Spanish family [31] and in one from Finland [32], both with cases of ovarian cancer. Finally, we found an in frame deletion in exon 20 in a large family. More detailed data about this deletion will be reported elsewhere (manuscript in preparation).

### Deleterious *BRCA2* mutations

Analysis of the *BRCA2* gene revealed 12 distinct germ line mutations with predicted deleterious effects on the BRCA2 protein in 20 families (Table 4). Among these pathogenic mutations 5 have not yet been reported in the Breast Cancer Information Core, BIC. Thus, the novel truncating mutation c.4030_4035delinsC (p.Asn1344Hisfs), shared by 6 families, is *BRCA2* most frequent mutation in our series and accounts for 30% of the *BRCA2* mutations. In the eleven carriers identified, a total of ten breast cancers, three ovarian cancers and one squamous oesophagus cancer have been diagnosed. Median age at diagnosis was 48 for breast (range 32–74) and 52 for ovarian cancer (range 48–60). Clinic and pathological characteristics of tumors from individuals carrying this mutation are shown in Table 5.

**Table 4:** **Germ line *BRCA2 *pathogenic mutations in breast and ovarian cancer families from Asturias**

**Exon**	**BIC nomenclature**	**HGVS nomenclature**	**Predicted effect**	**N families**	**Families origen**	**Comments**
3		c.262_263delCT	p.Leu88Alafs	1	Andalucía	Novel
10	2041insA	c.1813dupA	p.Ile605Asnfs	2	Asturias	Worldwide
11		c.2095 C>T	p.Gln699*	3	Asturias	Novel
11	3058A>T	c.2830A>T	p.Lys944*	1	Dominican Republic	Worldwide
11		c.3263dupC	p.Gln1089Serfs	1	Albacete	Novel
11		c.4030_4035delinsC	p.Asn1344Hisfs	6	Asturias	Novel
11	4859insA	c.4631dupA	p.Asn1544Lysfs	1	Asturias	Europe
11	5344del4	c.5116_5119delAATA	p.Asn1706Leufs	1	Castilla/León	Castilla/León, Founder
11	5804del4	c.5576_5579delTTAA	p.Ile1859Lysfs	1	País Vasco	País Vasco
18		c.8042_8043delCA	p.Thr2681Serfs	1	Andalucía	Novel
23	9254del5	c.9026_9030delATCAT	p.Tyr3009Serfs	1	Cantabria	Cataluña, Founder
25	9538delAA	c.9310_9311delAA	p.Lys3104Valfs	1	Castilla/León	Castilla/León, Founder

**Table 5:** **Clinic pathological characteristics of tumors from individuals carrying the mutation: *BRCA *2 4258_4263delinsC (p.Asn1344Hisfs)**

**Family.individual**	**Sex**	**Age**	**Tumor**	**Histopathology**	**Stage**	**Grade**	**ER**	**PgR**	**HER2**
27.1	F	52	Br	IDC	I	3	Neg	Neg	-
		60	Ov	Serous	IC	-	-	-	-
27.2	F	32	Br	IDC	IIB	3	Neg	Neg	Pos
27.3	M	60	Es	Squamous	III	-	-	-	-
208.1	F	50	Br	ILC	ND	-	-	-	-
		57	Br	IDC	ND	-	-	-	-
208.2	F	74	Br	-	IIIB	-	-	-	-
331.1	F	48	Ov	Serous	IV	3	-	-	-
331.2	F	49	Br	ILC	I		Pos	Pos	Neg
424.1	F	50	Ov	Serous	IC	2	-	-	-
609.1	F	43	Br	IDC	IIIA	-	Pos	Pos	Neg
		43	Br	DCIS		-	-	-	-
795.1	F	42	Br	IDC	IIA	-	Pos	Pos	-
795.2	F	39	Br	IDC	IV	-	Pos	Pos	-

Age age of cancer diagnosis, F female, M male, Br breast cancer, Ov ovarian cancer, Es esophagus cancer, IDC invasive ductal carcinoma, ILC invasive lobular carcinoma, DCIS ductal carcinoma in situ. ER estrogen receptor status, PR progesterone receptor status, Her2 Her2Neu status, - not applicable or no data, Neg negative, Pos positive.

The second most frequent novel mutation in *BRCA2* was c.2095 C>T (p.Gln699*) identified in three Asturian families, while the following novel truncating mutations were found in one family each: c.262_263delCT (p.Leu88Alafs); c.3263dupC (p.Gln1089Serfs); and c.8042_8043delCA (p.Thr2681Serfs). Individuals with these 3 novel mutations come either from Andalucía (Southern Spain) or from Albacete (Eastern Spain) where, to our knowledge, a comprehensive mutational analysis has not been reported yet.

Eight families carried 7 previously reported *BRCA2* mutations. Two families carried the mutation c.1813dupA (p.Ile605Asnfs), which is very common in Western Europe, mostly in Germany where a possible founder effect has been suggested [33]. The other 6 known mutations were found in one family each. The c.5116_5119delAATA (p.Asn1706Leufs) and c.9310_9311delAA (p.Lys3104Valfs) mutations, recurrent in Castilla-León, were found in two families originally from that area [34]. The pathogenic mutation c.5576_5579delTTAA (p.Ile1859Lysfs) was found in one family from the País Vasco (Northern Spain) and has been previously described in other Spanish populations [13,35]. Another family carried the frame-shift mutation c.4631dupA (p.Asn1544Lysfs), which has also been described in Western European populations [36]. The frame-shift c.9026_9030delATCAT (p.Tyr3009Serfs), which is a founder mutation originating in the Northeast of Spain [37], was identified in one family from Cantabria (Northern Spain). Finally, an African American woman with early onset bilateral breast cancer carried the nonsense mutation c.2830A>T (p.Lys944*), which has 6 entries in the BIC database and has been found in a Swedish series [38] but has not been previously reported in the Spanish population.

In contrast to the large *BRCA1* genomic deletions discussed above, no large genomic rearrangements were found to affect *BRCA2* in any of the families included in our study.

### Unclassified variants

Twenty two different variants of unknown significance were identified in a total of 29 affected index cases, 3 of which had not been previously reported, 2 in *BRCA1* and 1 in *BRCA2* (Table 6). The *BRCA1* missense mutation c.287A>C (p.Asp96Ala) has no BIC records, although the mutation p.Asp96Asn in the same position is considered pathogenic by some authors due to the fact that this amino acid is fully conserved throughout evolution [39]. We could not perform co-segregation studies, as the only first degree relative with cancer was deceased at the time of the study. The second novel *BRCA1* variant is the c.656A>T (p.Asp219Val) found in a family with 6 breast cancers. Two bioinformatics tools, *Sorting Intolerant From Tolerant* (SIFT) [40] and *Polymorphism Phenotyping* (PolyPhen) [41] indicate that this variant may alter the function of the protein. However, this variant does not segregate with the disease in this family and the affected residue is not conserved in mammals. A novel *BRCA2* variant found in the present work is the missense mutation c.6847C>A (p.Pro2283Thr), affecting a conserved residue in chordates. According to the above bioinformatics tools, this variant could be also pathogenic but it did not segregate with the disease in a family with two ovarian cancers. Consequently, these two variants are probably non-pathogenic.

**Table 6:** ***BRCA1 *and *BRCA2 *variants of unknown significance detected in breast and ovarian cancer families from Asturias**

**Gene**	**Exon**	**BIC nomenclature**	**HGVS nomenclature**	**Predicted effect**	**Nº families**	**Reference**
*BRCA1*	3	234T>A	c.115T>A	p.Cys39Ser	1	BIC, LOVD
	5	318G>T	c.199G>T	p.Asp67Tyr	2	BIC, LOVD
	6		c.287A>C	p.Asp96Ala	1	Novel
	10		c.656A>T	p.Asp219Val	1	Novel
	11	1186A>G	c.1067A>G	p.Gln356Arg	1	BIC, LOVD
	11	4158A>G	c.4039A>G	p.Arg1347Gly	1	BIC, LOVD
	15	4654G>T	c.4535G>T	p.Ser1512Ile	1	BIC, LOVD
	16	5075A>A	c.4956G>A	p.Met1652Ile	2	BIC, LOVD
	17	5173C>T	c.5054C>T	p.Thr1685Ile	1	BIC, LOVD
	19	5273G>T	c.5154G>T	p.Trp1718Cys	2	BIC, LOVD
	21	IVS20-6_IVS20-4del	c.5278-6_5278-4del	No effect	1	Campos et al. [42]
	22	IVS21-8C>T	c.5333-8C>T	Unkown	1	BIC
*BRCA2*	3	IVS2-T7>A)	c.68-7T>A	p.Asp23_Leu105del	2	BIC, LOVD
	10		c.1714G>A	p.Val572Ile	1	UMD-BRCA2
	11	6110G>A	c.5882G>A	p.Ser1961Asn	1	BIC
	11	6328C>T	c.6100C>T	p.Arg2034Cys	2	BIC, LOVD
	11	6976A>G	c.6748A>G	p.Thr2250Ala	1	BIC, LOVD
	11		c.6847C>A	p.Pro2283Thr	1	Novel
	18		c.8023A>G	p.Ile2675Val	1	UMD-BRCA2, LOVD
	20	8731T>C	c.8503T>C	p.Ser2835Pro	2	BIC, LOVD
	22	9078G>T	c.8850G>T	p.Lys2950Asn	1	BIC, LOVD
	27	10204A>T	c.9976A>T	p.Lys3326*	2	BIC, LOVD

UMD-BRCA2: http://www.umd.be/BRCA2/.

LOVD, BRCA1: http://chromium.liacs.nl/LOVD2/cancer/home.php?select_db=BRCA1.

LOVD, BRCA2: http://chromium.liacs.nl/LOVD2/cancer/home.php?select_db=BRCA2.

While the remaining variants have been previously reported, controversies persist in the literature regarding the pathogenicity of some of them. This is the case of missense mutation c.115T>A (p.Cys39Ser), which is registered as a variant of unknown significance in the BIC database, but alters the protein structure and is considered deleterious by some authors [39,43]. We found it in a family with breast and ovarian cancer, but it did not segregate with the disease as it was not present in one of the sisters who developed breast cancer at the age of 33. On the other hand, two *BRCA1* missense variants, c.5054C>T (p.Thr1685Ile) and c.5154G>T (p.Trp1718Cys), affecting highly conserved amino acid residues, have been predicted to be deleterious by a number of studies, including a multifactorial likelihood-ratio model [44], evolutionary conservation analyses and functional assays [39,45,46]. In our population, we could not study co-segregation of those variants, but the number of coincident results in favor of causality suggests that these variants can be deleterious.

We found the nonsense mutation c.9976A>T (p.Lys3326*) in two unrelated families. In spite that this mutation introduces a stop codon, causing premature termination of the protein, it is considered in BIC as with no clinical significance. In agreement with these observations, this mutation does not segregate with the disease in one of the families studied in this work. Some other variants are probably non-pathogenic, with odds of >100:1 in favor of neutrality according to multifactorial model by Easton et al. [44]. This can be the case of *BRCA1* c.199G>T (p.Asp67Tyr) and *BRCA2* c.6748A>G (p.Thr2250Ala) and c.8850G>T (p.Lys2950Asn).

### Geographic distribution of two novel recurrent mutations

Asturias is a Northern Spain community of 1 million inhabitants, bordered by Galicia to the West, Cantabria to the East and Castilla-León to the South. It has been rather isolated from the rest of Spain by its high mountains and it was one of the few areas that never came under the Muslim control that lasted in most of the Iberian Peninsula for five centuries. Although modern society has made possible migration phenomena, small geographically isolated communities still remain within deep valleys in a rural environment.

**Figure 1:**
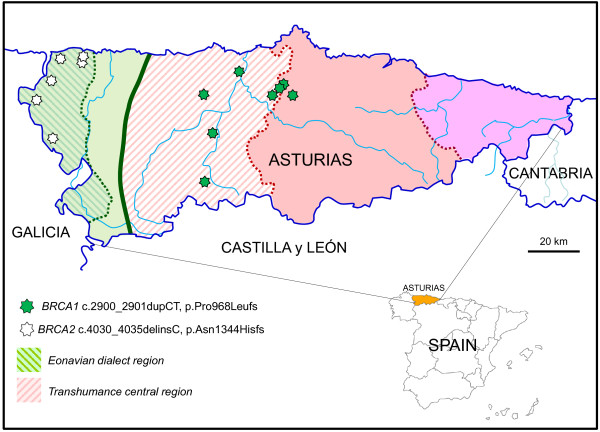
**Map of Asturias showing the geographical origin of the families with the two most frequent novel *BRCA1 *and *BRCA2 *mutations.** Two main linguistic areas are separated by a solid line while dialect boundaries are separated by dotted lines (based in map created with data from dialectologists Zamora-Vicente and Fernandez-Rei). *BRCA1* c.2900_2901dupCT mutation can be related to a transhumance population established in the vicinities of a Roman pathway of historical importance while c.4030_4035delinsC *BRCA2* is closely associated to Eonavian dialect population.

The origin of the eight families with the c.2900_2901dupCT (p.Pro968Leufs) *BRCA1* novel mutation clustered along a region extending from the Asturias coastline to the mountains bordering the province of Leon to the South (Figure 1). An historical important roman pathway (*Calzada de la Mesa*) communicating these two regions is found in the vicinity. This is the area where *Vaqueiros*, a seasonal transhumance population were established. With different habits and their own dialect, they have been for centuries a distinct social group, suggesting a possible founder effect of this mutation.

Regarding the other recurrent mutation c.4030_4035delinsC (p.Asn1344Hisfs) in *BRCA2*, the six families with this mutations were from a small area situated on the western border of Asturias (Figure 1), geographically isolated by two deep river valleys (Eo and Navia rivers) running from South to North. Interestingly, the population living there has their own and distinct dialect called Eonavian confirming the isolation and thus suggesting a founder effect of this mutation.

## Conclusions

In this study we conducted for the first time a comprehensive *BRCA1/2* screening in a group of 256 high risk families living in Asturias. We found that 59 families carried a pathogenic mutation, 39 in *BRCA1* and 20 in *BRCA2*, 8 of them being novel. Two mutations were found in families who cluster in two geographical and customary isolated areas, suggesting a founder effect, although future haplotype analysis would be necessary to confirm this hypothesis. Besides, all the families who carried the previously reported Galician founder mutations had Galician ancestors. In total, these three mutations plus a known recurrent mutation account for approximately 50% of all affected families, being 60% of *BRCA1* and 30% of *BRCA2* mutations recurrent. The results of the present study suggest that Asturias belongs to the group of geographical areas in which a small numbers of mutations account for a large proportion of *BRCA1*/2 mutations.

Consequently, testing a person from these areas for their respectively recurrent mutations before sequencing the complete genes could be a cost and time-efficient way to assess if the individual has *BRCA1* or *BRCA2* germ line mutations. Finally, we believe that conducting a specific mutational analysis on unselected cases of both breast and ovarian cancer for the Asturian women whose origin are these two small areas where the novel mutations seem to cluster, may be helpful and effective in cancer prevention terms.

## Competing interests

The authors declare that they have no competing interests.

## Authors’ contributions

PB, MB: designed the study, analyzed clinical and mutational data and drafted the manuscript. ISR, ASP, MGA, AL: performed mutational analyses and MLPA studies. ML, YF: revised clinical and pathological data. JMPF: revised the mutational data and helped to draft the manuscript. AP: completed data collection of families’ origins. All authors approved the final manuscript.

## Pre-publication history

The pre-publication history for this paper can be accessed here:

http://www.biomedcentral.com/1471-2407/13/243/prepub
